# The Gulstonian Lectures for 1842, on the Mutual Relation between Anatomy, Physiology, Pathology, and Therapeutics, and the Practice of Medicine

**Published:** 1842-07

**Authors:** 


### Art. XVII.

*The Gulstonian Lectures for* 1842, *on the Mutual Relation between Anatomy, Physiology, Pathology and Therapeutics, and the Practice of Medicine.* By Marshall Hall, m.d. f.r.s. &c.—*London,* 1842. 8vo, pp. 86. With Three Plates.

That the subject of these Lectures was well chosen, no one can hesitate to admit; and that few of the present generation have surpassed the lecturer in the successful application of physiological truth to the explanation of pathological phenomena, will also, we think, be now generally conceded. Yet we must take the liberty of expressing some degree of disappointment in regard to the mode in which he has treated his subject; for the greater part of the lectures consist of familiar illustrations of well-known principles, such as appear to us better adapted for the instruction of students than for the edification of the learned body to whom they were addressed. We doubt not, however, that Dr. Hall was guided, in his selection of topics, by his estimate of the previous education possessed by his auditors, and of their general habits of thought; and of this we find an obvious indication in the introductory remarks, which contain what we should have scarcely thought necessary in *these* days, a defence of the general principle, that no one can be a sound practitioner, whether of physic or surgery, who is not also conversant with the leading facts of anatomy and physiology.

Nevertheless, like all that Dr. Hall writes, these lectures contain many acute ideas and interesting suggestions; but these are not unmingled with what we deem grave errors. We shall briefly notice the chief of each class.

The first lecture is devoted to Physiology; the second to Pathology; and the third to Therapeutics. The first topic considered is the relative importance of the functions of ingestion and egestion; and Dr. Hall arrives at what we deem the very just conclusion, that, startling as it may *primâ facie* appear, the functions of egestion are more immediately necessary to the maintenance of life than those of ingestion. An animal may live for some time without food, and even without oxygen; but it is speedily killed if the excretion of carbonic acid by the lungs, or of urea by the kidney, is prevented. The interesting enquiry is suggested, whether the law of diffusion of gases (by which the interchange of oxygen and carbonic acid in the air-cells of the lungs is regulated,) will enable us to determine by what addition of carbonic acid to the atmospheric air, the exhalation of this gas from the blood may be entirely prevented. That it is the accumulation of carbonic acid in a limited atmosphere, rather than the withdrawal of oxygen, which is injurious to an animal confined in it, has been shown by the experiments of Dr. D. B. Reid; who found that if provision be made for withdrawing the carbonic acid from the air as fast as it is generated, (by means, for example, of a large surface of lime-water or solution of caustic potass,) the animal appeared to suffer but little inconvenience until the oxygen was nearly exhausted. The next of the chief subjects treated of in the first lecture, is the Physiology of the Circulation, in which Dr. Hall repeats his well-known views as to the entire dependence of the capillary and venous circulation upon the action of the heart; upon this question, however, we have too frequently stated the grounds of our entertaining a contrary opinion, for it to be requisite that we should now enter upon it. We shall only stop to remark, therefore, that Dr. Hall, in alluding to the supposed explanation of the circulation of *acardiac* fœtuses which was offered by Sir A. Cooper, does not seem to be aware that there is at least one case on record, in which, after the most careful examination by an accomplished anatomist, this explanation was found to be completely inapplicable. We may further point out that Müller, who is quoted by Dr. Hall as an important authority on his side, can scarcely be regarded as a consistent advocate of the doctrine; since whilst he states in one place that "the motion of the blood through the capillaries is effected solely by the action of the heart," he admits in several others, that the circulation may be greatly influenced by a local change in the part, *turgor vitalis* for instance; which is only another mode of saying that an increase of vital action in the part will accelerate the circulation through it, the very point upheld by Dr. Alison, Dr. Carpenter, and others who take the opposite side to Dr. Hall in this question. The coronary circulation is next dwelt upon in some detail; and it is shown that the union of the systemic and pulmonic hearts into one mass obviates the necessity, which would otherwise exist, for a special distribution of vessels to the right side. In fishes, in which the whole heart is pulmonic, and in which the trunk that issues from it is to be regarded as a pulmonary artery (conveying venous blood) rather than as an aorta, the coronary vessels arise, as we are informed by Dr. Grant, not from that trunk, but from the vessels that return arterial blood from the gills and send it to the system. The flow of blood into the coronary arteries is synchronous, not with the systole of the heart, but with its diastole: the flow is prevented by the contraction of the muscular structure, which is seen to render the heart pale so long as it lasts, and is occasioned by the subsequent reaction of the aorta. Dr. Hall thinks that the injection of blood into the tissue of the heart is a principal cause of its dilatation; and supposes that some variation in the arrangement of the arteries of the auricles and ventricles occasions their alternate action and regular rhythm. This idea, however, appears to us quite inconsistent with the well-known fact, that the hearts of cold-blooded animals will continue to act regularly long after they have been entirely drained of their blood. Dr. Hall subsequently makes some very just remarks upon the necessity of a due supply of arterial blood for the action of the nervous centres; but we think that he over-rates its importance in regard to the muscular system, since, as we have just seen, certain muscles will act for many hours without it.

In the second lecture, one of the subjects most dwelt upon is Asphyxia; and an interesting parallel is drawn between the gaspings and other violent abnormal efforts which are witnessed during suffocation, and the phenomena of death from loss of blood. Dr. Hall points out that, in both instances, the actions are probably not of a *reflex* character, as are those of ordinary respiration, but are *centric*, having their origin in the disturbance of the supply of blood to the medulla oblongata. He subsequently adverts to Secondary Asphyxia, or death from convulsion at a period more or less remote from that at which the cause of asphyxia was operating, as by no means an unfrequent result of accident and disease; and points out the necessity of continuing for some time our efforts to restore the blood to its normal state. The influence of various forms of interrupted circulation in the production of congestions, extravasations of blood, dropsies, &c. is then ably pointed out; and the effect of a suspension of the coronary circulation in producing sudden death is much dwelt upon, perhaps rather too much. This last question Dr. Hall proposes to investigate by experiment; and until it shall have been determined, we think it better not to speculate upon it. Our own impression is, that a mere suspension of the coronary circulation will produce rather a gradual than a sudden failure of the heart's action. Several other interesting and important subjects are treated of in this lecture; but there are none which present any peculiar novelty.

In the third lecture we may especially direct attention to the remarks upon the Action of Poisons, (the investigation of which is very important to the pathologist from its connexion with that of ordinary medicinal agents,) as giving what we believe to be the true view of the subject. Dr. Hall considers that the view of those who advocate the doctrine of the transmission of all poisons by the vascular system, and of those who attribute their operation to the nervous system, are alike erroneous if *exclusively* adopted. The experiments of Mr. Blake and others have clearly proved that the local action of poisons on parts remote from those to which they are applied, is to be attributed *in many instances* to their absorption and actual transmission by the blood-vessels; but, as Dr. Hall justly remarks, there are many agents which indubitably act on the incident nerves, and which occasion phenomena analogous to those resulting from poisonous agents. He instances hydrophobia and tetanus as examples of the occurrence of two sets of parallel morbid phenomena, the former resulting from the reception of a poison into the blood, the latter from an irritation conveyed through an incident nerve.

We quite agree with Dr. Hall, (Preface, p. viii.) that "a treatise on the subject of these lectures, in which each principle should be set forth by a *fact*, an *observation*, or an *experiment*, would be of incalculable value to pupils." Such a treatise Dr. Hall has long projected; and we hope it will not be much longer ere he will be able to execute it.

---

